# Pre-Spondylectomy Radiofrequency Ablation for Spinal Chordoma: An Illustrative Case and Systematic Review of Clinical Applications and Outcomes

**DOI:** 10.3390/curroncol33090569

**Published:** 2026-09-20

**Authors:** Holden Husbands, Drake Johnston, Matthan Tharakan, Ahmed Hussan, Kishore Balasubramanian, Patrick Bettiol, Benjamin Chou, Sanaa Hameed, Zachary A. Smith, M. Burhan Janjua, John F. Burke, Hakeem J. Shakir

**Affiliations:** 1 Department of Neurosurgery, University of Oklahoma Health Sciences Center, Oklahoma City, OK 73104, USA; holden-husbands@ou.edu (H.H.); drake-johnston@ou.edu (D.J.); matthan-tharakan@ou.edu (M.T.); ahmed-hussan@ou.edu (A.H.); kishore-balasubramanian@ou.edu (K.B.); benjamin-chou@ou.edu (B.C.); sanaa-hameed@ou.edu (S.H.); zachary-smith@ou.edu (Z.A.S.); muhammad-janjua-1@ou.edu (M.B.J.); john-burke@ou.edu (J.F.B.); 2 Department of Orthopedic Surgery, University of Oklahoma Health Sciences Center, Oklahoma City, OK 73104, USA; patrick-bettiol@ou.edu

**Keywords:** chordoma, spine, sacrum, radiofrequency ablation, cryoablation, laser interstitial thermal therapy

## Abstract

Chordomas are rare, locally aggressive spinal tumors. En bloc resection may improve local control but can cause substantial morbidity. Thermal ablation may provide a less invasive adjunct or alternative in selected cases. We systematically reviewed reports of radiofrequency ablation (RFA), cryoablation, laser interstitial thermal therapy, high-intensity focused ultrasound, and similar thermal techniques for spinal or sacral chordoma to characterize their clinical applications and reported clinical and radiographic outcomes across pre-resection, primary non-operative, and palliative settings. Eligible studies reported at least one ablation-related clinical or radiographic outcome. Data were descriptively synthesized, and risk of bias was assessed using the Joanna Briggs Institute criteria. Thirteen studies including 33 patients and 34 ablated sites were identified. Cryoablation was used in 21 patients, RFA in 11 patients, and laser interstitial thermal therapy in one patient. Ablation was used as a pre-resection adjunct, primary treatment for unresectable disease, or palliation for recurrent or metastatic lesions. Most reports described pain improvement, short-term local stability, and few major complications. We also report an L4 chordoma treated with CT-guided RFA and vertebroplasty before staged en bloc spondylectomy, without a new neurologic deficit or local recurrence at 18 months. Thermal ablation may be a technically feasible adjunct, primary non-operative option, or palliative strategy for carefully selected spinal and sacral chordomas. In the presented mobile-spine case, planned pre-spondylectomy RFA was feasible before staged en bloc resection; however, limited follow-up and sparse heterogeneous literature preclude conclusions regarding long-term local control, recurrence reduction, survival, or routine use. Ablation should not replace oncologically appropriate en bloc resection when that procedure can be safely achieved and should be considered selectively within multidisciplinary chordoma care.

## Simple Summary

A chordoma is a tumor that occurs within the spine that is difficult to manage due to its aggressive tendency to invade other bones in the surrounding area. The rarity of chordoma has made the data that does exist inconsistent and has resulted in a variety of recommendations for how to treat and monitor these patients. The different presentations of the tumor across these cases also make real recommendations difficult. Given the variety of treatments that do currently exist, however, we attempted to catalog and understand how they may be applied in treatment. In addition, we used a case report from our medical center to present a novel way of attacking these tumors. The review describes how ablation has been applied and summarizes reported pain, neurologic, radiographic, and complication outcomes. Available evidence is limited and heterogeneous; therefore, thermal ablation should be considered a selective adjunct or palliative option rather than a replacement for oncologically appropriate resection.

## 1. Introduction

Chordoma is a rare, locally invasive tumor of notochordal origin, typically arising from the skull base or spine, and carries a poor prognosis due to high recurrence rates and difficulty achieving complete eradication [1,2]. Chordomas are often resistant to radiotherapy and chemotherapy because of their dual epithelial–mesenchymal differentiation and local invasion of critical neurovascular structures [1,2]. Management of spinal chordoma is challenging, and en bloc spondylectomy remains the gold standard for local control and long-term survival in appropriately selected patients, often combined with adjuvant therapies [3,4]. Despite resection of the tumor and surrounding healthy tissue, this approach can be associated with major blood loss, infection, wound complications, and functional impairment, depending on tumor size and location [4,5,6]. En bloc resection followed by adjuvant radiotherapy has been associated with longer local recurrence-free survival (median 35 vs 14 months) and improved overall survival compared with intralesional resection alone [6]. In a meta-analysis of 562 patients, photon therapy achieved local control rates of 97%, 92%, 88%, 77%, and 71% at 1, 2, 3, 5, and 7 years, respectively, with overall survival rates of 100%, 90%, 89%, 86%, and 80% at the same time points [7]. However, comparisons across treatment strategies are limited by small cohorts and retrospective designs.

Literature describing palliative and definitive thermal ablation has emerged as a novel adjunctive or stand-alone option for spinal and sacral chordomas, with reported roles in pain control, local debulking, and local control of unresectable or recurrent tumors using radiofrequency ablation (RFA), cryoablation, laser interstitial thermal therapy (LITT), photon therapy, and high-intensity focused ultrasound (HIFU) [8,9,10,11,12,13,14,15,16,17,18,19]. These modalities can be delivered percutaneously under image guidance or intraoperatively as local adjuvants, providing a minimally invasive means to treat bulky or anatomically constrained lesions while attempting to preserve surrounding structures and limit morbidity compared with more destabilizing approaches [8]. There is therefore a need to expand adjunctive strategies that improve quality of care and provide additional options in complex chordoma cases. In this context, this report presents percutaneous image-guided RFA of a mobile-spine vertebral chordoma followed by staged en bloc spondylectomy and systematically reviews published clinical reports of thermal ablation for spinal and sacral chordoma. The objectives of the systematic review were to characterize the clinical applications of thermal ablation across pre-resection, primary non-operative, and palliative settings and to summarize reported imaging-based local outcomes, pain, neurologic or functional outcomes, complications, and follow-up at treated sites. To our knowledge, this is the first detailed report of staged percutaneous pre-spondylectomy RFA before multistage en bloc resection of a mobile-spine chordoma. This approach is presented as a patient-specific technical adjunct in a favorable anatomic setting, rather than as evidence supporting routine ablation for chordoma.

## 2. Presentation of Case

**Figure 1:**
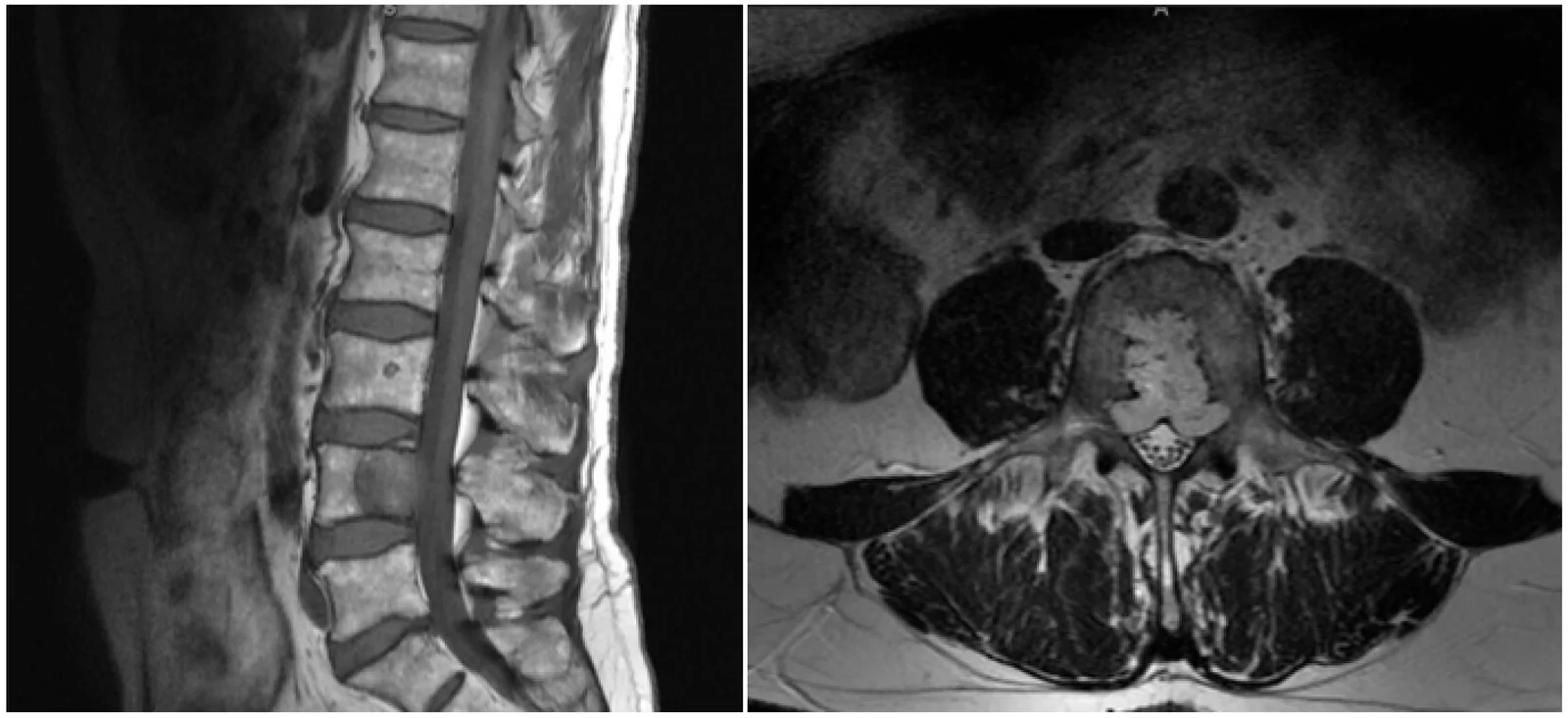
Preoperative MRI Lumbar Spine Sagittal and coronal planes.

A 66-year-old gentleman with a history of left lower extremity radiculopathy and back pain beginning in July 2023, with worsening symptoms, was referred to our facility in January 2024. MRI in February 2024 confirmed a destructive soft tissue mass involving the posterior L4 vertebral body lesion (Figure 1). An initial CT-guided biopsy yielded cytokeratin-positive carcinoma cells but was considered inconclusive, prompting a repeat image-guided biopsy combined with ablative treatment. Symptoms included lower back pain, radiculopathy on the left side, constipation, and pelvic pain. The patient underwent CT-guided biopsy with concurrent RFA of the L4 lesion, and pathologic analysis confirmed chordoma. Following this procedure, a staged two-part en bloc L4 spondylectomy was planned and performed in mid-April 2024.

### 2.1. Surgical Technique

Under general anesthesia in the angiography suite, bilateral transpedicular access to L4 was obtained using fluoroscopic guidance, and multiple fresh and frozen core specimens were taken before any ablation to ensure adequate diagnostic tissue. Once pathology confirmed sufficient sampling, radiofrequency probes were advanced into the anterior vertebral body under 3D imaging software and fluoroscopic guidance, and RF ablation was performed at 95 °C for 12 min with irrigation, followed by the removal of intravertebral debris using a hand drill. Vertebroplasty was then carried out through the same pedicular tracts: bone plugs were placed and freshly made bone cement was injected under continuous fluoroscopy, carefully restricted to the anterior two thirds of the body to avoid posterior leakage. The trocars were disengaged from the cement cast and removed. Estimated blood loss was <50 cc, and there were no intraoperative or periprocedural complications. For the second stage of the planned en bloc spondylectomy, imaging confirmed the operative site. Under general anesthesia, the patient was positioned prone on a Jackson table. After subperiosteal dissection exposed the spinous processes, laminae, facets, and transverse processes, the L2/L3 cranial facet capsules were carefully preserved. Instrumentation was placed using anatomic landmarks from L2–S1 (excluding L4); bilateral pedicle screws were inserted, and bilateral iliac screws were inserted after exposure and preparation of the posterior superior iliac spine (PSIS). The final construct spanned L2–S1, with carbon-fiber screws at L3 and L5 for future tumor surveillance. Multilevel laminectomy (L3–L5) was undertaken using standard instruments, with lateral extension for complete decompression. Bilateral L4 pediculectomy was performed, and after removal of L4 transverse processes, anterolateral accesses to the thecal sac enabled L3–4 and L4–5 diskectomies. Dural sealant was applied to L4 pedicles to minimize the risk of tumor spread. Circumferential dissection around L4 freed the vertebral body, and the posterior longitudinal ligament (PLL) was resected at L3–4 and L4–5 disk spaces. The L4 vertebral body was found to be completely mobile, unattached posteriorly. Alloderm was placed between L4 and the ventral dura for thecal sac protection pending anterior resection. Main rods were placed in the screw tulips from L2 to the pelvis and cantilever reduction was completed. A satellite rod system was added for additional stability. Irrigation, final decortication, and morselized allograft for fusion augmentation followed. A sub-fascial drain was placed. Calcium sulfate beads with antibiotics were placed to provide sustained infection prophylaxis. The surgical wound was closed in a multilayered fashion. The patient was transferred to recovery, with an estimated blood loss of 1000 cc, in a stable condition and without complication. Two days later the patient was transported to the OR and placed supine under general anesthesia. The abdomen was prepared and draped in a sterile fashion. Vascular/abdominal surgical access was obtained via a low midline incision, facilitating anterior exposure of the spine from L4 to the L5–S1 ventral disk space. Meticulous dissection was performed to mobilize vascular and soft tissue structures, allowing safe corridor to the ventral lumbar spine. L5–S1 diskectomy was then achieved using a combination of straight and angled curettes. Fluoroscopy was utilized to confirm the working trajectory and ensure thorough decompression. With disk material removed and cartilaginous endplates prepared using straight curettes and sequential sizers, a PEEK lordotic cage, packed with allograft, was implanted at L5–S1 via indirect fluoroscopic guidance. Shims were inserted to secure the cage, achieving restoration of disk height and bilateral neural foraminal decompression. Attention was then directed to the en bloc corpectomy of L4. Diskectomy at L3–4 and L4–5 was then performed from the ventral approach, followed by careful mobilization and removal of the L4 vertebral body, preserving key anterior and posterior structures as planned. Hemostasis was maintained throughout. A carbon-fiber corpectomy cage, pre-packed with allograft, was contoured and deployed into the defect to allow MRI surveillance postoperatively. Precise cage placement restored sagittal alignment and provided stable anterior column support for the construct. Anterior spinal instrumentation was secured to augment fixation across the construct. Final alignment and cage positioning were confirmed with intraoperative fluoroscopy. Layered closure was accomplished in routine fashion by the access surgeon following copious irrigation of the field. The patient tolerated the procedure well, with no intraoperative complications. The patient was in stable condition, with no complications, and an estimated blood loss of 750 cc, and was then transferred to the recovery unit.

### 2.2. Follow-Up and Treatment

**Figure 2:**
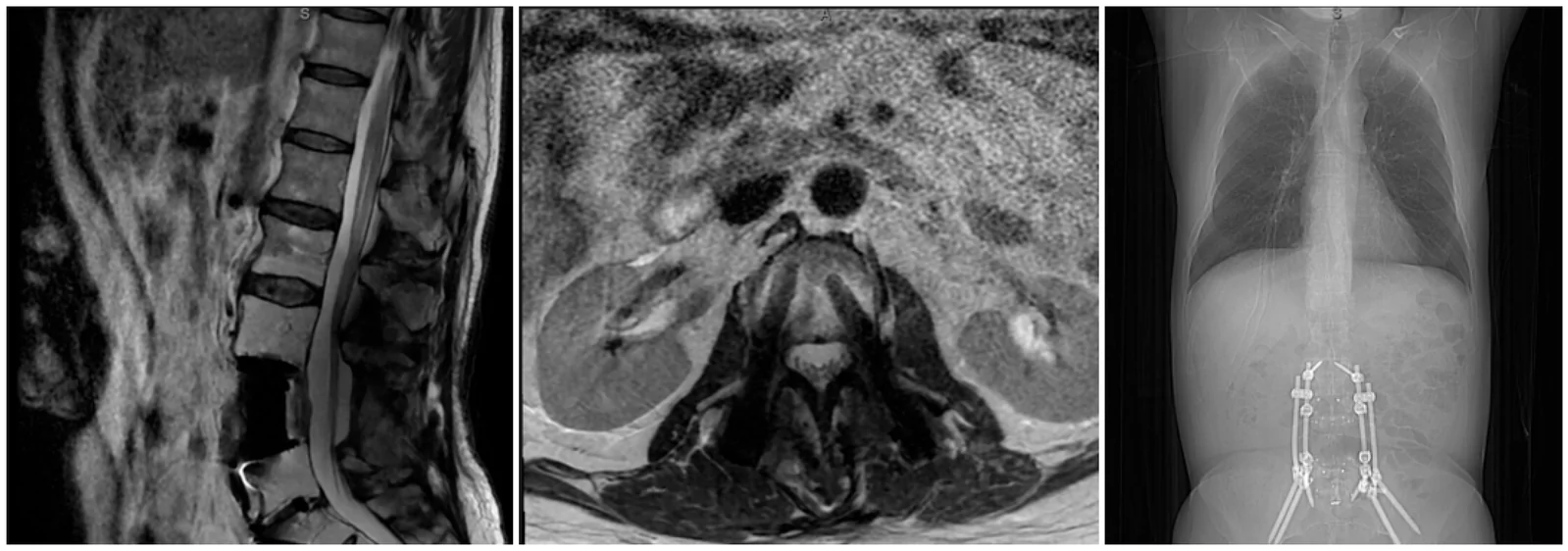
Postoperative MRI Lumbar Spine Sagittal, axial, with additional X-ray LRFS at 18 months follow-up.

Postoperatively, the patient was hemodynamically stable, with no weakness. The patient was discharged to an inpatient rehabilitation facility on postoperative day 7. At the 6-month and 1-year follow-up, MRI demonstrated no evidence of local recurrence, and the patient was clinically stable without acute distress. He reported residual mild pain and paresthesia in both lower extremities, which were effectively managed with the use of gabapentin. At the most recent 18-month follow-up, MRI demonstrated no evidence of local recurrence. This duration of follow-up provides only a short-term assessment and is insufficient to determine long-term oncologic control (Figure 2).

## 3. Materials and Methods

**Figure 3:**
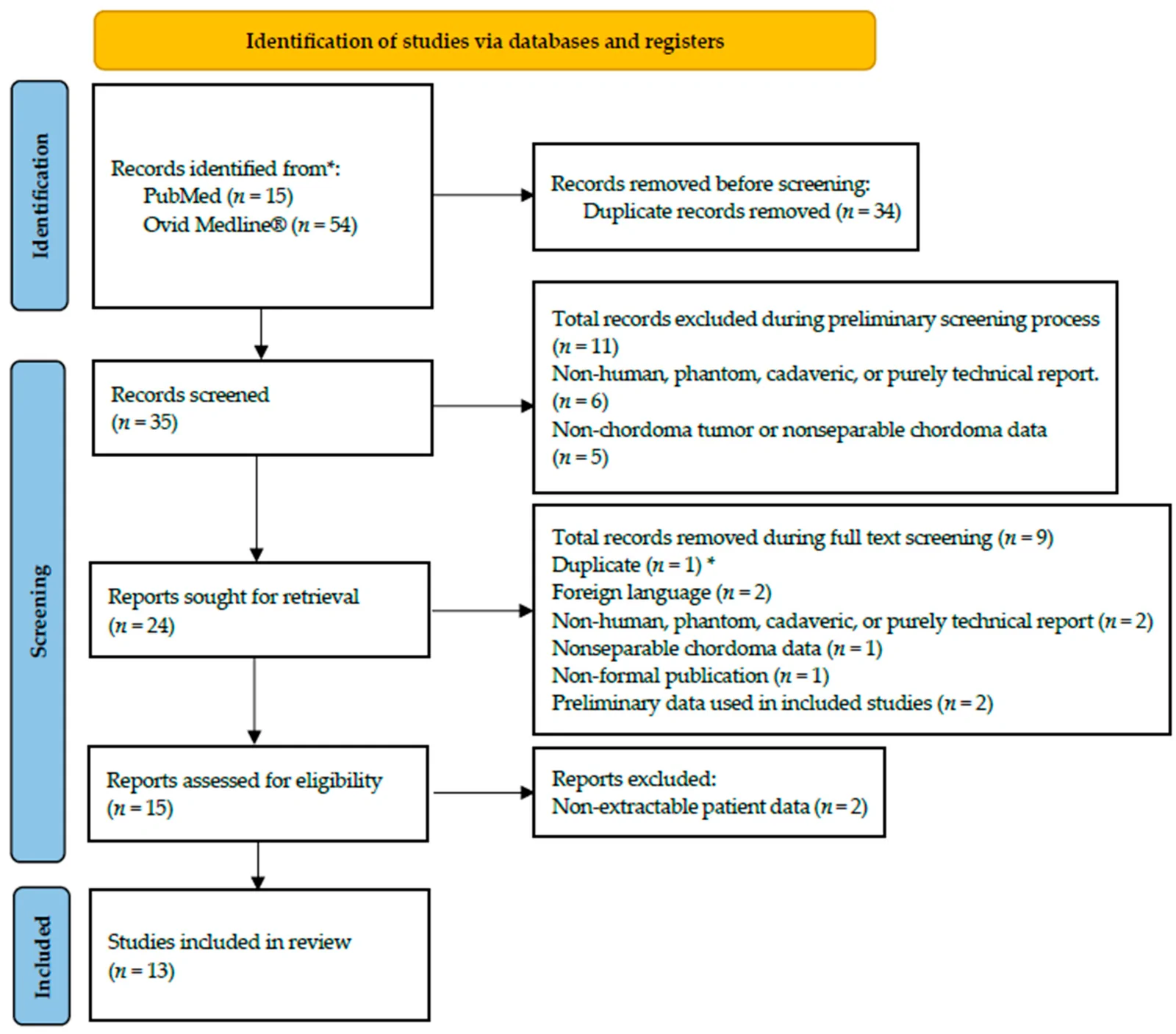
PRISMA flowchart for systematic review. Systematic review was conducted according to PRIMSA guidelines. * Study was separated in screening and found to have been already in consideration listed under different publishing author name (Cherix et al.) [14].

We conducted a systematic review of published clinical studies describing thermal ablation for spinal and sacral chordoma, reported in accordance with the Preferred Reporting Items for Systematic Reviews and Meta-Analyses (PRISMA) 2020 guidance [20] (Table S1). The review included case reports, case series, and small observational cohorts describing thermal ablation for histologically confirmed spinal or sacral chordoma, including sacrococcygeal and spinal metastatic sites. The review protocol was prospectively registered in the International Prospective Register of Systematic Reviews (PROSPERO; CRD420261430171) and received no external funding. The objective of the review was to characterize the clinical applications and technical approaches of thermal ablation and to summarize reported clinical and radiographic outcomes across three settings: pre-resection or intraoperative adjunctive treatment, primary non-operative treatment of unresectable disease, and palliative treatment of recurrent or metastatic disease. We searched PubMed (MEDLINE via PubMed) and MEDLINE via Ovid from 2000 to February 2026 using a Boolean strategy combining terms for chordoma, spinal or sacral location, and thermal ablation. A representative MEDLINE search was: (chordoma OR chordomas OR “Chordoma” [MeSH Terms]) AND (spine OR spinal OR vertebral OR vertebra* OR sacrum OR sacral OR sacrococcygeal) AND (ablation OR ablate* OR cryoablation OR cryotherapy OR “radiofrequency ablation” OR RFA OR “laser interstitial thermal therapy” OR LITT OR microwave OR “thermal therapy”). Similar strategies were applied to Embase, and reference lists of included studies were screened for additional eligible reports. Studies were included if they met all of the following criteria: (1) histologically confirmed chordoma involving the mobile spine, sacrum, or sacrococcygeal region, including spinal metastases; (2) use of thermal ablation (RFA, cryoablation, LITT, HIFU, or similar) directed at the chordoma lesion; and (3) reporting of at least one prespecified outcome at the treated site, including imaging-based local outcomes (stability, partial response, progression, recurrence, or progression-free status), pain response, neurologic or functional outcome, ablation-related complications, follow-up duration, or need for subsequent local therapy. Studies were excluded if (1) the tumor type was not chordoma or chordoma-specific data could not be isolated; (2) the lesion was skull base only without spinal or sacral ablation; (3) only non-thermal interventions were described; or (4) no patient-level outcomes were reported (e.g., purely technical or preclinical studies). Search results were imported into a reference manager and Rayyan software for deduplication and screening. Titles and abstracts were screened to identify reports likely to describe chordoma in a spinal or sacral location treated with thermal ablation; non-chordoma, non-spinal/sacral, or non-ablation studies were excluded. First, bibliographic duplicates were removed before preliminary screening; of the 69 records, 34 were removed as duplicates. During preliminary screening, 11 total records were excluded, 6 of which were non-human, phantom, cadaveric, or purely technical reports and 5 of which were studies of non-chordoma tumors or studies without separable chordoma data. The remaining 24 reports were subject to full text review, with the removal of 9 reports due to factors not assessable during preliminary screening. These reports included 1 previously unrecognized duplicate that was indexed under a different author name, 2 foreign language reports, 1 non-formal publication, and 2 preliminary reports whose patient data was subsequently reported in studies included for review, and 1 report without separable chordoma data. The same exclusion category may therefore appear more than once at different stages of the screening process, but no individual record was counted more than once, as summarized in the PRISMA flow diagram (Figure 3). Palliative studies were included because symptom relief, neurologic or functional change, procedure-related complications, and imaging-based local outcomes are clinically relevant outcomes for patients in whom curative-intent resection is not feasible or would be associated with unacceptable morbidity. Two reviewers independently assessed risk of bias and reporting quality using Joanna Briggs Institute Critical Appraisal Checklists for case reports and case series, according to study design. Discrepancies were resolved by discussion and, when necessary, consultation with a third reviewer (K.B.). Two reviewers (H.H. and D.J.) independently abstracted data onto standardized forms. Extracted variables included: (1) study characteristics (first author, year, design); (2) number of patients and lesions or ablated sites; (3) patient demographics and key comorbidities; (4) disease state at the treated site (primary/unresectable, recurrent, metastatic); (5) tumor level and anatomic extension; (6) ablation modality and technical details (RFA, cryoablation, LITT, or other; guidance modality; intraoperative vs staged vs palliative use); (7) presenting signs and symptoms; (8) concurrent or staged surgery and adjuvant therapies; and (9) outcomes, including pain and neurologic change, local control or recurrence at the ablated site, complications, and follow-up duration and imaging modality. Because all included studies were retrospective with heterogeneous reporting, we summarized data using counts and proportions and did not calculate pooled time to event metrics or comparative local control rates. The prespecified primary outcomes were local radiographic behavior at the treated site, pain response, neurologic or functional outcomes, and ablation-related complications; secondary outcomes were follow-up duration, need for additional local therapy, and perioperative parameters when ablation was used before surgery. For the case report we reviewed a patient who underwent pre-spondylectomy ablation at the University of Oklahoma Health Sciences Center in March 2024. Extracted data included age, sex, body mass index, comorbidities, and prior oncologic history, as well as operative indications, operative time, and chronic disease history. We recorded spine-related complications, presenting symptoms, imaging findings, and the interval between ablation and en bloc spondylectomy. Surgical management variables included ablation modality, use of spondylectomy, and length of hospital stay. Perioperative and postoperative outcomes included complication types (e.g., infection, neuropathic pain, recurrence), need for reoperation, and antibiotic use. Follow-up data captured revision surgery, persistent sequelae, and mortality.

## 4. Results

The systematic review included 13 studies comprising 33 patients and 34 ablated sites. The included evidence consisted of case reports, small case series, one retrospective cohort, and one early clinical trial describing thermal ablation of spinal or sacral chordoma lesions. Patient, tumor, treatment, and outcome data are summarized in Table 1 and Table 2.

### 4.1. Patient and Tumor Characteristics

Among 33 patients with available demographic data, the mean age was 57.04 years, with males (*n* = 14, 42.4%), females (*n* = 15, 45.5%), and four patients (12.1%) with unspecified sex. Pre-ablation tumor diameter was reported for 24 treated lesions, with a mean diameter of 4.85 cm. Tumor volume was reported in eight studies but could not be compared quantitatively because measurement methods and units were inconsistent. Across 34 ablated sites, tumors were most commonly located in the sacrum (primary sacral lesions *n* = 16/34, 47.1%) and sacrococcygeal region (*n* = 10/34, 29.4%), followed by ischiorectal fossa (*n* = 4/34, 11.8%), cervical spine (*n* = 2/34, 5.9%), acetabulum (*n* = 1/34, 2.9%), and iliac wing (*n* = 1/34, 2.9%). Local invasion data were available for 24 lesions and demonstrated frequent sacral foraminal involvement (*n* = 9/24, 37.5%) and adjacent bowel proximity (*n* = 4/24, 16.6%), with additional instances of sciatic nerve compression (*n* = 2/24, 8.2%), nerve root involvement (*n* = 2/24, 8.3%), paravertebral dural sac compression, pelvic invasion, C7 paraspinal soft tissue extension, acetabular involvement, pararectal/ischiosciatic extension, pudendal artery involvement, and thigh/perianal/spine base extension (each *n* = 1/24, 4.2%). At the site level (*n* = 40 disease state entries), primary/unresectable lesions accounted for 21 (52.5%), recurrent disease for 18 (45.0%), and metastatic disease for 1 (2.5%).

### 4.2. Ablation and Treatment Characteristics

Three main ablation modalities were used: cryoablation in 21 of 33 patients (63.6%), RFA in 11 of 33 (33.3%), and LITT in 1 of 33 (3.0%). Guidance was predominantly CT-based, with selected use of MRI guidance and MRI with thermography for LITT, and extracorporeal high-intensity focused ultrasound in the Gillies series. Clinical context at the treated site included pre-resection adjunct use (e.g., intraoperative or staged ablation) in selected cervical and sacral cases, primary non-operative treatment for unresectable sacral/sacrococcygeal chordomas, and palliative or post-recurrence treatment for recurrent or metastatic disease, including metastatic C7 spinous lesions and heavily pretreated sacral tumors. Many patients had undergone prior surgery and/or radiation before ablation, whereas others in the primary cryoablation series were treated nonoperatively with or without adjuvant radiotherapy or stereotactic radiosurgery. (Table 1 and Table 2).

### 4.3. Reported Clinical, Imaging, and Safety Outcomes

Reporting of local control and recurrence was heterogeneous, but across the 13 studies, most ablated sites with short- to intermediate-term follow-up remained radiographically stable or demonstrated partial response at the treated level, particularly in primary cryoablation and palliative RFA series. Pain relief was commonly described, with many patients experiencing a meaningful reduction in lumbosacral, sciatica, pelvic, or coccygeal pain and associated improvement in mobility or limb function when such outcomes were reported. Ablation-related complications were infrequent and generally transient, including occasional nerve root palsy or systemic symptoms such as malaise and oliguria, while most patients in the cohort had no reported ablation-specific complications described during the follow-up period (Table 1). Interpretation of local-control and oncologic outcomes was limited by heterogeneous indications, variable imaging surveillance, inconsistent definitions of progression or local control, and short or incompletely reported follow-up across studies. Reported outcomes differed according to treatment intent. In palliative studies, pain relief, functional or neurologic change, procedure-related complications, and short-term imaging-based local outcomes were the principal reported endpoints. In pre-resection reports, technical feasibility and the ability to proceed with planned surgery were most relevant, whereas primary non-operative reports primarily described symptom change and imaging-based local outcomes during available follow-up.

## 5. Discussion

This illustrative case describes pre-spondylectomy, image-guided RFA followed by staged en bloc resection for a mobile-spine chordoma. In combination with a systematic review of published thermal ablation reports, it provides a descriptive synthesis of how thermal ablation has been used in selected clinical settings and of the clinical and radiographic outcomes reported in the available literature. However, neither the present case nor the available literature establishes improved long-term local control, reduced recurrence risk, survival benefit, or a routine role for RFA in chordoma treatment. Chordomas remain difficult to treat, with a 5-year overall survival of approximately 50% and an incidence of only eight cases per million per year, representing 1–4% of primary bone malignancies [3,5,6,22]. Most published series are small and retrospective, limiting opportunities to refine technique and compare strategies. Although en bloc spondylectomy is the current standard for achieving durable local control when feasible, it carries substantial morbidity and does not obviate the need for additional modalities that may improve long-term outcomes and quality of life.

Our case highlights a planned strategy using image-guided RFA before en bloc resection. In this setting, ablation may alter intravertebral tumor tissue and facilitate operative handling; however, its effect on surgical margins, tumor viability at the margin, blood loss, or long-term oncologic control cannot be determined from this case. Ablation may alter tissue characteristics and facilitate operative handling in selected cases; however, its independent effect on dissection, preservation of adjacent structures, surgical margins, or blood loss has not been established. In this patient, acceptable perioperative blood loss, absence of major perioperative complications or new neurologic deficits, and no radiographic local recurrence at 18 months support the technical feasibility of the staged approach; however, they do not establish long-term oncologic benefit. Together with prior reports, our findings suggest that thermal ablation can serve as a useful adjunct rather than a replacement for oncologic resection, particularly when operative fields are anatomically complex or heavily pretreated. Importantly, this report should not be interpreted as supporting routine use of preoperative RFA for all mobile-spine chordomas. En bloc resection with oncologically appropriate margins remains the preferred treatment when it can be safely accomplished. The potential value of thermal ablation is most relevant in carefully selected cases in which anatomy, tumor extension, proximity to neural, vascular, or visceral structures, concern regarding difficult intraoperative manipulation, prior treatment, unresectability, or anticipated operative morbidity complicates conventional management. In such settings, ablation may be considered as an individualized adjunct after multidisciplinary review; it is not a substitute for adequate oncologic resection where wide or margin-appropriate resection remains safely achievable. In our systematic review of 13 studies including 33 patients, thermal ablation was applied across three main clinical contexts: pre-resection adjunct, primary non-operative management of unresectable sacral/sacrococcygeal disease, and palliation of recurrent or metastatic lesions. Most treated sites were sacral or sacrococcygeal with extension into the pelvis, foramina, and surrounding soft tissues, consistent with the axial distribution and locally aggressive behavior of chordoma. Slightly more than half of treated sites were primary or unresectable lesions, with the remainder predominantly recurrent and a small minority metastatic, reflecting the high local recurrence burden and occasional distant spread. The pattern of invasion included sacral foramina, adjacent bowel, sciatic nerve and nerve roots, paravertebral dural sac, pelvic structures, and paraspinal soft tissues. This helps explain the profound symptom burden and technical complexity of achieving disease control while preserving bowel, bladder, sexual, and lower extremity function. The technical applicability of ablation varies substantially by anatomic site. Sacral and thoracic chordomas may involve neural foramina, spinal cord, nerve roots, bowel, pelvic organs, and major vessels, potentially increasing the risk of neurologic and functional morbidity and constraining achievable ablation margins. The predominantly intravertebral L4 lesion in the illustrative case permitted bilateral transpedicular access and differs meaningfully from more extensive sacral, thoracic, skull-base, or extraosseous disease. Accordingly, thermal ablation does not eliminate the fundamental challenge of achieving adequate local disease control in anatomically constrained chordoma and must be individualized according to tumor location, extension, and treatment intent. Three principal ablative modalities were used: cryoablation (63.6% of patients), RFA (33.3%), and LITT (3.0%), with one small series employing extracorporeal HIFU [8,9,10,14]. Cryoablation series, typically using CT guidance and argon–helium systems, leveraged predictable iceball geometry to conform ablation volumes around sacrococcygeal tumors while protecting nearby neural and visceral structures, demonstrating feasibility in unresectable or heavily pretreated disease. RFA was used both percutaneously and intraoperatively; for example, intraoperative RFA has been applied to a giant cervical paravertebral chordoma to shrink and firm the mass and achieve tumor-free exposed margins before gross total excision. LITT was reported in a single metastatic C7 spinous process lesion arising from a sacral chordoma, used palliatively under MRI and thermography guidance. HIFU offered a fully extracorporeal approach for advanced sacral chordoma, illustrating an additional route for thermal cytoreduction without new incisions, although not in a pre-spondylectomy setting. Despite heterogeneous reporting and variable follow-up, most case reports and series described clinically meaningful improvements in pain and function after ablation [8,10,11,12,13]. Lumbosacral, sciatic-type, pelvic, and coccygeal pain frequently decreased, and some patients experienced improved ambulation, reduced claudication, or better limb function. Many lesions remained radiographically stable or showed partial response at short- to intermediate-term follow-up, particularly in unresectable sacrococcygeal tumors treated with cryoablation and in palliative RFA or HIFU series, suggesting that thermal ablation may provide short- to intermediate-term symptom relief and favorable short- to intermediate-term imaging-based local outcomes in selected settings. Additional data from other malignancies have shown pain reduction of 62.5% at 24 h and 80.9% at 6 months after ablation, supporting a broader analgesic role for these techniques [23]. Ablation-related complications were uncommon and generally transient, including isolated nerve root palsy and systemic symptoms such as malaise and oliguria, while most patients had no procedure-specific complications. The available literature does not establish that thermal ablation improves long-term oncologic outcomes in chordoma. Follow-up was limited and inconsistently reported, and most studies lacked standardized definitions of local control, comparative cohorts, or survival analyses. Accordingly, the observed short-term radiographic stability, partial response, and symptom improvement should be interpreted as descriptive signals of technical feasibility and potential palliative benefit rather than evidence of reduced recurrence risk, durable disease control, or survival advantage. Within this context, our staged L4 case represents a novel extension of thermal ablation from palliative or intraoperative adjunct toward a deliberately planned pre-spondylectomy maneuver in the mobile spine. Previous reports have described intraoperative RFA or cryoablation as local adjuvants during open resection of mobile-spine and sacral chordomas, or percutaneous ablation for unresectable sacral disease and spinal metastases [2,9]. In contrast, our patient underwent percutaneous CT-guided RFA with vertebroplasty, followed days later by staged posterior release and instrumentation and subsequent anterior en bloc L4 spondylectomy. Among the 13 studies and 33 patients identified, we found no prior description of percutaneous, image-guided ablation of a mobile-spine vertebral chordoma performed as a temporally staged step before multistage en bloc spondylectomy. Performing ablation through the biopsy tract may also reduce tumor cell seeding along the needle path; one series of 82 chordoma patients reported surgical seeding in 7.3% of cases [24]. In this context, the staged L4 case represents a technical extension of thermal ablation toward a planned pre-spondylectomy maneuver in a carefully selected mobile-spine lesion. Percutaneous CT-guided RFA with vertebroplasty was followed by posterior release and instrumentation and subsequent anterior en bloc L4 spondylectomy. Although ablation may have altered tumor consistency or facilitated handling in this case, its independent contribution to operative blood loss, surgical margins, tumor-cell dissemination, recurrence risk, or long-term oncologic control cannot be determined. The case should therefore be interpreted as demonstrating feasibility rather than efficacy, and it should not be generalized to all chordoma locations or clinical contexts [25,26].

This study has several limitations. The systematic review includes a small cohort of retrospective case reports and case series that are vulnerable to selection, publication, and reporting biases and lack standardized outcome definitions or control groups. Follow-up intervals, imaging modalities, and criteria for local control or progression varied widely, precluding robust survival analyses and limiting conclusions regarding long-term durability. The pooled cohort is modest and heterogeneous with respect to clinical indication, tumor location, disease extent, prior treatment, and ablation technique. The available evidence does not permit determination of whether ablation reduces recurrence risk, improves survival, facilitates wider surgical margins, decreases blood loss, or provides benefit beyond oncologically appropriate resection alone. In addition, the illustrative L4 case reflects a single carefully selected patient at one institution, with 18 months of follow-up. The favorable short-term result supports technical feasibility but cannot establish long-term oncologic efficacy or generalizability. These findings should therefore be regarded as descriptive and hypothesis-generating rather than definitive evidence supporting routine ablation for chordoma.

## 6. Conclusions

Thermal ablation has been reported across pre-resection, primary non-operative, and palliative clinical settings for spinal and sacral chordoma. In carefully selected patients, it may serve as a technical adjunct to surgery, an option for unresectable disease, or a palliative intervention aimed at symptom relief and favorable short-term radiographic behavior. In the presented L4 case, staged percutaneous RFA before en bloc spondylectomy was technically feasible and was associated with an acceptable short-term clinical and radiographic outcome. However, this observation does not establish reduced recurrence risk, long-term local control, survival benefit, improved surgical margins, reduced blood loss, or a routine role for preoperative ablation. When oncologically appropriate en bloc resection can be safely achieved, ablation is not necessarily required and should not replace definitive surgery. Treatment decisions should be individualized through specialized multidisciplinary evaluation and should account for tumor location, local extension, relationships to critical structures, prior therapy, treatment goals, anticipated morbidity, and feasibility of adequate local disease control. Larger prospective registries and multi-institutional studies are needed to define patient selection, technical parameters, integration with surgery and radiotherapy, and long-term oncologic outcomes.

## Figures and Tables

**Table 1:** Overview of included studies.

Study	Year	Study Type	Tumor Size	Number of Patients	Age (Mean, Years)	Sex (M/F)	Tumor Location	Disease State	Ablation Type	Clinical Application/Treatment Intent	Guidance Modality	Surgery Type/Margin	Outcomes and Follow-Up
Anis et al. [19]	2004	Case Series	15 cm (M), 10 cm (F)	2	72, 65	1M 1F	Sacrum	Recurrent	RFA	Palliative	CT	Prior Resections only	Pain: Improved, Functional response: Improved, Imaging response: Responded, Follow-up: 7–8 mos, Procedure-related complication: Oliguria/Malaise (resolved), Subsequent surgery/Radiotherapy: Not reported
Teichgraber et al. [16]	2006	Case report	N/A	1	66	F	Sacrococcygeal	Recurrent	RFA	Pre-resection, intraoperative	MRI	Partial ablations	Pain: Improved, Functional response: Improved, Imaging response: Responded, Follow-up: 4 mos, Procedure-related complication: rectal perforation, Subsequent surgery/Radiotherapy: Not reported
Marchal et al. [18]	2006	Case Series		1	49	M	Sacrum	Recurrent	RFA	Pre-resection, intraoperative	CT	Total ablation	Pain: Improved, Functional response: Improved, Imaging response: Responded, Follow-up: 1 mos, Procedure-related complication: Fistula hemorrhage, Subsequent surgery/Radiotherapy: None reported
Kurup et al. [10]	2012	Retrospective	14–39 mm	5	31–80 (mean 58)	3M 2F	Sacrococcygeal, iliac wing, acetabulum	Recurrent	Cryoablation	Post-recurrence/palliative	CT (*n* = 5), US/MR (*N* = 1)	N/A	Pain: Improved, Functional response: No change, Imaging response: stable disease, Follow-up: every 3 mos for 9 mos total, Procedure-related complication: Pain at ablation site, Subsequent surgery/Radiotherapy: None reported
Hamamoto et al. [13]	2014	Case Report	N/A	2	64, 68	1M 1F	Sacrum	Recurrent	RFA	Palliative	CT	Previous Resections Only	Pain: Improved, Functional response: Improved, Imaging response: Responded, Follow-up: 1 yr, Procedure-related complication: N/A, Subsequent surgery/Radiotherapy: None reported
Williams et al. [15]	2017	Case report	N/A	1	75	F	C7 spinous (metastasis), sacrum (primary)	Metastatic	LITT	Palliative, metastatic	MRI/thermography	None	Pain: Improved, Functional response: Improved, Imaging response: Responded, Follow-up: 3 mos, Procedure-related complication: None reported, Subsequent surgery/Radiotherapy: None reported
Gillies et al. [11]	2017	Case Series	P1; 464.7 cm^3^, P2: 91.4 cm^3^, P3: 975.15 cm^3^, P4: 2769.8 cm^3^	4	33–73 (mean 51.75)	N/A	Sacrum	Primary (2)/ recurrent (2)	RFA	Primary, non-operative; intraoperative	CT	N/A	Pain: N/A, Functional response: Improved, Imaging response: Responded, Follow-up: 6 mos, Procedure-related complication: Bladder dysfunction (*n* = 2), Subsequent surgery/Radiotherapy: None reported
Zhou et al. [17]	2018	Case Report	3.67 × 3.83 × 1.6 cm (→3.23 × 3.46 × 0.8 cm post-RFA)	1	40	M	C4 vertebral body, C3-C6 mass	Primary, unresectable	RFA	Pre-resection, intraoperative	Direct Visualization	Gross total–tumor free	Pain: N/A, Functional response: N/A, Imaging response: responded, Follow-up: 3,6,12,18,24 mos + annually, Procedure-related complication: c5 nerve palsy (resolved), Subsequent surgery/Radiotherapy: None reported
Pipola et al. [12]	2018	Case report/ Review	35 × 30 × 8 cm	1	64	F	Sacrum	Recurrent	Cryoablation	Pre-resection, intraoperative	MRI	N/A	Pain: N/A, Functional response: Mixed, Imaging response: Responded, Follow-up: 6 mos, Procedure-related complication: worsened fecal incontinence, Subsequent surgery/Radiotherapy: None reported
Inaba et al. [9]	2019	Case report	20 mm	1	64	M	Sacrum	Recurrent	Cryoablation	Primary, Non-operative	CT	Not applicable	Pain: Improved, Functional response: Improved, Imaging response: Responded, Follow-up: 3–6 mos yearly for 4 yrs, Procedure-related complication: Dysuria, Subsequent surgery/Radiotherapy: None reported
Li et al. [8]	2020	Case Series	7.8–12.5 cm	9	35–65 (mean 53.67)	3M 6F	Sacrococcygeal, S1–S3	Primary, unresectable	Cryoablation	Primary, non-operative	CT	None	Pain: Improved, Functional response: Improved, Imaging response: recurrence, Follow-up: 3 mos for 2 yrs then every 6 mos, Procedure-related complication: None, Subsequent surgery/Radiotherapy: None reported
Cherix et al. [14]	2021	Care Series	Mean 44.4 cm^3^ (median 10.5, range 0.5–146.6)	4	36–78 (58.5 mean)	3M 1F	Sacrum. coccyx	Recurrent (2)/ Primary (2)	Cryoablation	Primary, Non-operative	CT	Not applicable	Pain: Improved, Functional response: Improved, Imaging response: stable disease + recurrence, Follow-up: 5 yrs, Procedure-related complication: Subcutaneous emphysema, Subsequent surgery/Radiotherapy: None reported
Bostwick et al. [21]	2025	Clinical Trial	N/A	1	67.2	N/A	Sacrum	Unlisted, most likely primary	Cryoablation/immunotherapy infusion	Palliative, Metastatic	CT	N/A	Pain: N/A, Functional response: N/A, Imaging response: Stable Disease, Follow-up: N/A, Procedure-related complication: Skin rash at injection site, Subsequent surgery/Radiotherapy: None reported

**Table 2:** Summary of patient demographics and interventions.

Characteristics (No. of Patients for Whom Information Is Available)	Value (Among Patients with Available Data)
Cohort size (No.)	33 No. (%)
Gender (*n* = 33)	No. (%)
Male	14 (42.4%)
Female	15 (45.5%)
Not Specified	4 (12.1%)
Mean patient age (*n* = 33)	57.04 Years
Male	58.71 Years
Female	60.53 Years
Not Specified with age given	51.75 Years
Local invasion (*n* = 24)	No. (%)
Sciatic Nerve compression	2 (8.2%)
Paravertebral mass compressing Dural Sac	1 (4.2%)
Pelvic Involvement	1 (4.2%)
Adjacent Bowel	4 (16.6%)
Nerve roots	2 (8.3%)
Sacral foramina	9 (37.5%)
C7 Paraspinal Soft tissues	1 (4.2%)
Acetabulum	1 (4.2%)
Pararectal space/Ischosciatic nerve	1 (4.2%)
Pudendal artery	1 (4.2%)
Thigh/Perianal/Spine base	1 (4.2%)
Disease state (*n* = 32)	No. (%)
Primary/Unresectable	15 (46.9%)
Metastatic	1 (3.1%)
Recurrent	16 (50.0%)
Tumor locations Ablated (*n* = 34)	No. (%)
Sacrum (Primary)	16 (47.1%)
Sacrococcygeal	10 (29.4%)
Cervical	2 (5.9%)
Ischiorectal Fossa	4 (11.8%)
Acetabulum	1 (2.9%)
Iliac Wing	1 (2.9%)
Adjuvant Therapy (*n* = 33)	No. (%)
Radiofrequency	11 (33.3%)
Cryoablation	21 (63.6%)
Laser Interstitial Thermal Therapy	1 (3.0%)

## Data Availability

The original contributions presented in this study are included in the article/Supplementary Material. Further inquiries can be directed to the corresponding author.

## Supplementary Materials

The following supporting information can be downloaded at: https://www.mdpi.com/article/10.3390/curroncol33090569/s1, Table S1: PRISMA 2020 Checklist.

## Abbreviations

CT	Computed tomography
HIFU	High-intensity focused ultrasound
JBI	Joanna Briggs Institute
RFA	Radiofrequency Ablation
PRISMA	Preferred Reporting Items for Systematic Reviews and Meta-Analyses
MRI	Magnetic resonance Imaging
LITT	Laser Interstitial thermal therapy
