# Supplementary material for: Pre-Spondylectomy Radiofrequency Ablation for Spinal Chordoma: An Illustrative Case and Systematic Review of Clinical Applications and Outcomes
Source: Curr Oncol. 2026 Sep 20;33(9):569. doi: 10.3390/curroncol33090569 (PMC13605485; doi:10.3390/curroncol33090569)
Supplement: Supplementary file 1 [file curroncol-33-00569-s001.zip › curroncol-4522935-supplementary.pdf]

Table S1

PRISMA 2020 Checklist

Manuscript: Pre-Spondylectomy Radiofrequency Ablation for Spinal Chordoma: An Illustrative Case and Systematic Review

PROSPERO: CRD420261430171

PRISMA 2020 main checklist

Section and topic	Item #	Checklist item	Location where item is reported	Status
**TITLE**	**1**	Identify the report as a systematic review.	Title page, p. 1: "...An Illustrative Case and Systematic Review"	**Yes**
**ABSTRACT**	**2**	See the PRISMA 2020 for Abstracts checklist.	Abstract, pp. 3-4. Abstract checklist completed below. Funding and PROSPERO registration are not stated in the abstract.	**Yes**
**INTRODUCTION**	**3**	Describe the rationale for the review in the context of existing knowledge.	Introduction, pp. 5-6: chordoma biology and prognosis; limits of en bloc resection and radiotherapy; emerging thermal-ablation literature (refs 8-19); need for adjunctive strategies.	**Yes**
**INTRODUCTION**	**4**	Provide an explicit statement of the objective(s) or question(s) the review addresses.	Central Message, p. 2; last paragraph of Introduction, p. 6; Methods, Systematic Review, p. 10. Purpose is stated (evaluate thermal ablation across pre-resection, primary non-operative, and palliative indications). No explicit PICO review question.	**Yes**
**METHODS**	**5**	Specify the inclusion and exclusion criteria for the review and how studies were grouped for the syntheses.	Eligibility criteria, p. 11: histologically confirmed spinal/sacral/sacrococcygeal chordoma (including spinal metastases); thermal ablation (RFA, cryoablation, LITT, HIFU or similar); at least one ablation-related outcome. Exclusions: non-chordoma or non-isolable data; skull base only; non-thermal interventions; no patient-level outcomes. All eligible studies entered one descriptive synthesis (p. 13).	**Yes**
**METHODS**	**6**	Specify all databases, registers, websites, organisations, reference lists and other sources searched or consulted. Specify the date when each source was last searched or consulted.	Search Strategy, pp. 10-11: PubMed (MEDLINE), MEDLINE via Ovid, Embase, and reference lists of included studies; date window 2000 to February 2026. Abstract also says "other databases." Last search date is not given per source; trial registers and grey literature are not listed.	**Yes**
**METHODS**	**7**	Present the full search strategies for all databases, registers and websites, including any filters and limits used.	Search Strategy, p. 11: representative MEDLINE Boolean string provided; "similar strategies were applied to Embase." Full Ovid MEDLINE and Embase strings, filters, and limits are not presented. Consider adding as Table S1.	**Yes**
**METHODS**	**8**	Specify the methods used to decide which studies met the inclusion criteria, including how many reviewers screened each record and each report retrieved, whether they worked independently, and any automation tools used.	Study selection, pp. 11-12: reference manager and Rayyan for de-duplication and screening; title/abstract then full-text review. Number of independent screeners and how disagreements were resolved at screening are not stated (resolution process is described only for risk of bias).	**Yes**
**METHODS**	**9**	Specify the methods used to collect data from reports, including how many reviewers collected data from each report, whether they worked independently, any processes for obtaining or confirming data from study investigators, and any automation tools used.	Data Abstraction and Synthesis, pp. 12-13: two reviewers (H.H. and D.J.) independently abstracted data onto standardized forms. Contacting study investigators is not described.	**Yes**

Section and topic	Item #	Checklist item	Location where item is reported	Status
**METHODS**	**10a**	List and define all outcomes for which data were sought. Specify whether all results compatible with each outcome domain were sought, and if not, the methods used to decide which results to collect.	Data Abstraction and Synthesis, p. 13: pain and neurologic change; local control or recurrence at the ablated site; complications; follow-up duration and imaging modality. Handling of multiple measures/time points within a study is not specified.	**Yes**
**METHODS**	**10b**	List and define all other variables for which data were sought (e.g. participant and intervention characteristics, funding sources). Describe any assumptions made about missing or unclear information.	Data Abstraction and Synthesis, p. 13: study characteristics; patient/lesion counts; demographics; disease state; tumor level/extension; ablation modality and technical details; concurrent surgery/adjuvant therapy. Funding of included studies and missing-data assumptions are not described.	**Yes**
**METHODS**	**11**	Specify the methods used to assess risk of bias in the included studies, including the tool(s) used, how many reviewers assessed each study, whether they worked independently, and any automation tools used.	Risk of Bias, p. 12: Joanna Briggs Institute Critical Appraisal Checklists for case reports and case series; two reviewers independently; discrepancies resolved by discussion or a third reviewer (K.B.).	**Yes**
**METHODS**	**12**	Specify for each outcome the effect measure(s) (e.g. risk ratio, mean difference) used in the synthesis or presentation of results.	Data Abstraction and Synthesis, p. 13: counts and proportions; no pooled time-to-event metrics, comparative local-control rates, or other effect estimates.	**Yes**
**METHODS**	**13a**	Describe the processes used to decide which studies were eligible for each synthesis.	Methods, Systematic Review and Data Abstraction, pp. 10 and 13: all studies meeting eligibility entered a single descriptive synthesis; no grouping for quantitative synthesis.	**Yes**
**METHODS**	**13b**	Describe any methods required to prepare the data for presentation or synthesis, such as handling of missing summary statistics or data conversions.	Data Abstraction and Synthesis, p. 13: heterogeneous retrospective reporting; no data conversions or pooled time-to-event preparation. Missing-data handling is not specified.	**Yes**
**METHODS**	**13c**	Describe any methods used to tabulate or visually display results of individual studies and syntheses.	Results, p. 13: "Patient, tumor, treatment, and outcome data are summarized in Tables 1 and 2." Figure 1 is the PRISMA flow diagram (caption, p. 24). Tables 1 and 2 are cited but are not present in this .docx.	**Yes**
**METHODS**	**13d**	Describe any methods used to synthesise results and provide a rationale for the choice(s). If meta-analysis was performed, describe the model(s), heterogeneity methods, and software.	Data Abstraction and Synthesis, p. 13: descriptive synthesis using counts and proportions because studies were small, uncontrolled, and heterogeneous. Explicitly no meta-analysis.	**Yes**
**METHODS**	**13e**	Describe any methods used to explore possible causes of heterogeneity among study results (e.g. subgroup analysis, meta-regression).	Data Abstraction and Synthesis, p. 13: "no meta-analysis, pooled time-to-event analysis, comparative effect estimates, or formal subgroup analyses were performed."	**N/A**
**METHODS**	**13f**	Describe any sensitivity analyses conducted to assess robustness of the synthesised results.	Data Abstraction and Synthesis, p. 13: none performed (no quantitative synthesis).	**N/A**
**METHODS**	**14**	Describe any methods used to assess risk of bias due to missing results in a synthesis (arising from reporting biases).	Not reported as a methods item. Publication and reporting bias are noted qualitatively in Limitations, p. 22.	**No**
**METHODS**	**15**	Describe any methods used to assess certainty (or confidence) in the body of evidence for an outcome.	Not reported (no GRADE or equivalent certainty assessment).	**No**
**RESULTS**	**16a**	Describe the results of the search and selection process, from the number of records identified to the number of studies included, ideally using a flow diagram.	Results, p. 13: 13 studies, 33 patients, 34 ablated sites. Study selection, p. 12, and Figure 1 caption, p. 24, refer to a PRISMA 2020 flow diagram. Record-level counts (identified, duplicates, screened, full-text excluded) are not in the text, and Figure 1 is not embedded in this .docx.	**Yes**
**RESULTS**	**16b**	Cite studies that might appear to meet the inclusion criteria, but which were excluded, and explain why they were excluded.	Not reported. Full-text exclusions with reasons are not listed.	**No**
**RESULTS**	**17**	Cite each included study and present its characteristics.	Results, Patient and Tumor Characteristics and Ablation and Treatment Characteristics, pp. 13-15 (pooled). Individual reports are cited in Discussion (refs 8-19). Per-study characteristics are assigned to Tables 1 and 2, which are not in this file.	**Yes**

Section and topic	Item #	Checklist item	Location where item is reported	Status
**RESULTS**	**18**	Present assessments of risk of bias for each included study.	Methods state JBI appraisal was performed (p. 12). Results do not present per-study JBI judgments or a risk-of-bias table.	**No**
**RESULTS**	**19**	For all outcomes, present, for each study: (a) summary statistics for each group (where appropriate) and (b) an effect estimate and its precision, ideally using structured tables or plots.	Symptoms and Functional Outcomes, pp. 15-16: narrative pooled description of local control, pain, and complications. Per-study statistics and precision estimates are not shown in the text (Tables 1-2 cited). Comparative effect estimates are not applicable.	**Yes**
**RESULTS**	**20a**	For each synthesis, briefly summarise the characteristics and risk of bias among contributing studies.	Results, pp. 13-15, summarise design mix, demographics, sites, and modalities. Risk of bias among contributing studies is not summarised.	**Yes**
**RESULTS**	**20b**	Present results of all statistical syntheses conducted. If meta-analysis was done, present the summary estimate, precision, and heterogeneity.	No statistical/meta-analytic synthesis. Descriptive results are on pp. 13-16.	**N/A**
**RESULTS**	**20c**	Present results of all investigations of possible causes of heterogeneity among study results.	None performed (Methods, p. 13). Heterogeneity is discussed qualitatively in Results and Limitations.	**N/A**
**RESULTS**	**20d**	Present results of all sensitivity analyses conducted to assess robustness of the synthesised results.	None performed (Methods, p. 13).	**N/A**
**RESULTS**	**21**	Present assessments of risk of bias due to missing results (arising from reporting biases) for each synthesis assessed.	Not presented. Limitations, p. 22, mention publication and reporting bias qualitatively.	**No**
**RESULTS**	**22**	Present assessments of certainty (or confidence) in the body of evidence for each outcome assessed.	Not reported.	**No**
**DISCUSSION**	**23a**	Provide a general interpretation of the results in the context of other evidence.	Discussion, pp. 16-21, including Patient Selection, Systematic Review and Symptomatic Manifestations, Thermal Ablation Modalities, Clinical Outcomes, and Novelty of Treatment.	**Yes**
**DISCUSSION**	**23b**	Discuss any limitations of the evidence included in the review.	Limitations, pp. 21-22: small retrospective case reports/series; selection, publication, and reporting bias; no standardized outcomes or controls; heterogeneous follow-up and imaging; modest mixed cohort.	**Yes**
**DISCUSSION**	**23c**	Discuss any limitations of the review processes used.	Limitations, pp. 21-22, focus on the evidence base. Review-process limits (database coverage, language, independent screening, missing full search strings) are not separately discussed.	**Partial**
**DISCUSSION**	**23d**	Discuss implications of the results for practice, policy, and future research.	Patient Selection, pp. 17-18; Conclusion, pp. 22-23: selective adjunct within multidisciplinary care; not a substitute for en bloc resection; need for prospective registries and multi-institutional studies.	**Yes**
**OTHER INFORMATION**	**24a**	Provide registration information for the review, including register name and registration number, or state that the review was not registered.	Methods, Systematic Review, p. 10: PROSPERO CRD420261430171.	**Yes**
**OTHER INFORMATION**	**24b**	Indicate where the review protocol can be accessed, or state that a protocol was not prepared.	Registration number is given. Protocol URL/access statement is not provided.	**No**
**OTHER INFORMATION**	**24c**	Describe and explain any amendments to information provided at registration or in the protocol.	Not reported. If none, add: "No amendments were made to the registered protocol."	**No**
**OTHER INFORMATION**	**25**	Describe sources of financial or non-financial support for the review, and the role of the funders or sponsors in the review.	Title page, p. 2: "The authors received no financial support..." Methods, p. 10: review received no external funding. Role of funders is therefore not applicable.	**Yes**
**OTHER INFORMATION**	**26**	Declare any competing interests of review authors.	Title page, p. 2: no conflicts except Dr. John F. Burke, consultant for Highridge.	**Yes**
**OTHER INFORMATION**	**27**	Report which of the following are publicly available and where they can be found: template data collection forms; data extracted from	Not reported. MDPI Cancers also expects a Data Availability Statement.	**Yes**

Section and topic	Item #	Checklist item	Location where item is reported	Status
		included studies; data used for all analyses; analytic code; any other materials used in the review.		

PRISMA 2020 for Abstracts checklist

Section and topic	Item #	Checklist item	Location where item is reported	Status
**TITLE**	**1**	Identify the report as a systematic review.	Title, p. 1, includes "Systematic Review." Abstract heading does not restate this.	**Yes**
**BACKGROUND**	**2**	Provide an explicit statement of the main objective(s) or question(s) the review addresses.	Abstract Objective, p. 3, frames the clinical problem and ablation modalities. The structured review question is not stated as an objective.	**Yes**
**METHODS**	**3**	Specify the inclusion and exclusion criteria for the review.	Abstract Methods, p. 3: case reports, case series, observational cohorts of RFA, cryoablation, LITT, or similar for spinal/sacral chordoma with at least one ablation-related outcome. Exclusion criteria are not listed in the abstract.	**Yes**
**METHODS**	**4**	Specify the information sources used to identify studies and the date when each was last searched.	Abstract Methods, p. 3: MEDLINE, Embase, and other databases. Search dates are in Methods (2000-February 2026), not in the abstract.	**Yes**
**METHODS**	**5**	Specify the methods used to assess risk of bias in the included studies.	Abstract Methods, p. 3: Joanna Briggs Institute criteria.	**Yes**
**METHODS**	**6**	Specify the methods used to present and synthesise results.	Abstract Methods, p. 3: data summarized descriptively.	**Yes**
**RESULTS**	**7**	Give the total number of included studies and participants and summarise relevant characteristics of studies.	Abstract Results, pp. 3-4: 13 studies, 33 patients, 34 sites; age, sex, diameter, location, modality mix.	**Yes**
**RESULTS**	**8**	Present results for main outcomes, preferably indicating the number of included studies and participants for each. If meta-analysis was done, report the summary estimate and interval.	Abstract Results, p. 4: indications (neoadjuvant, primary non-operative, palliative), pain relief, local control, low complications; local-control/oncologic outcomes limited by short inconsistent follow-up. No meta-analysis.	**Yes**
**DISCUSSION**	**9**	Provide a brief summary of the limitations of the evidence included in the review.	Abstract Results and Conclusions, p. 4: limited follow-up, sparse heterogeneous literature, cannot conclude on long-term control, recurrence, survival, or routine use.	**Yes**
**DISCUSSION**	**10**	Provide a general interpretation of the results and important implications.	Abstract Conclusions, p. 4: technically feasible selective adjunct; should not replace oncologically appropriate en bloc resection.	**Yes**
**OTHER**	**11**	Specify the primary source of funding for the review.	Not in the abstract. Title page, p. 2, states no financial support.	**Yes**
**OTHER**	**12**	Provide the register name and registration number.	Not in the abstract. Methods, p. 10: PROSPERO CRD420261430171.	**Yes**

- This manuscript is a hybrid illustrative case plus systematic review. The checklist maps the systematic-review portion. The case (Presentation of Case through Follow-Up, pp. 6-10) is outside PRISMA but is the clinical frame for the review.
- Main checklist: 28 Yes, 1 Partial, 8 No, 5 N/A. Abstract checklist: 12 Yes, 0 Partial, 0 No.

From: Page MJ, McKenzie JE, Bossuyt PM, et al. The PRISMA 2020 statement: an updated guideline for reporting systematic reviews. BMJ. 2021;372:n71. Distributed under CC BY 4.0.
